# A theoretical framework for planar polarity establishment through interpretation of graded cues by molecular bridges

**DOI:** 10.1242/dev.168955

**Published:** 2019-02-01

**Authors:** Katherine H. Fisher, David Strutt

**Affiliations:** Department of Biomedical Science, University of Sheffield, Western Bank, Sheffield S10 2TN, UK

**Keywords:** Planar polarity, Planar cell polarity (PCP), Patterning, Gradient, Mathematical modelling, Asymmetry

## Abstract

Planar polarity is a widespread phenomenon found in many tissues, allowing cells to coordinate morphogenetic movements and function. A common feature of animal planar polarity systems is the formation of molecular bridges between cells, which become polarised along a tissue axis. We propose that these bridges provide a general mechanism by which cells interpret different forms of tissue gradients to coordinate directional information. We illustrate this using a generalised and consistent modelling framework, providing a conceptual basis for understanding how different mechanisms of gradient function can generate planar polarity. We make testable predictions of how different gradient mechanisms can influence polarity direction.

## Abstract (precis)

**Summary:** This Hypothesis uses a theoretical framework to explore how molecular bridges provide a general mechanism to interpret different forms of tissue gradients to establish planar polarity.

## Introduction

**Fig. 1.**
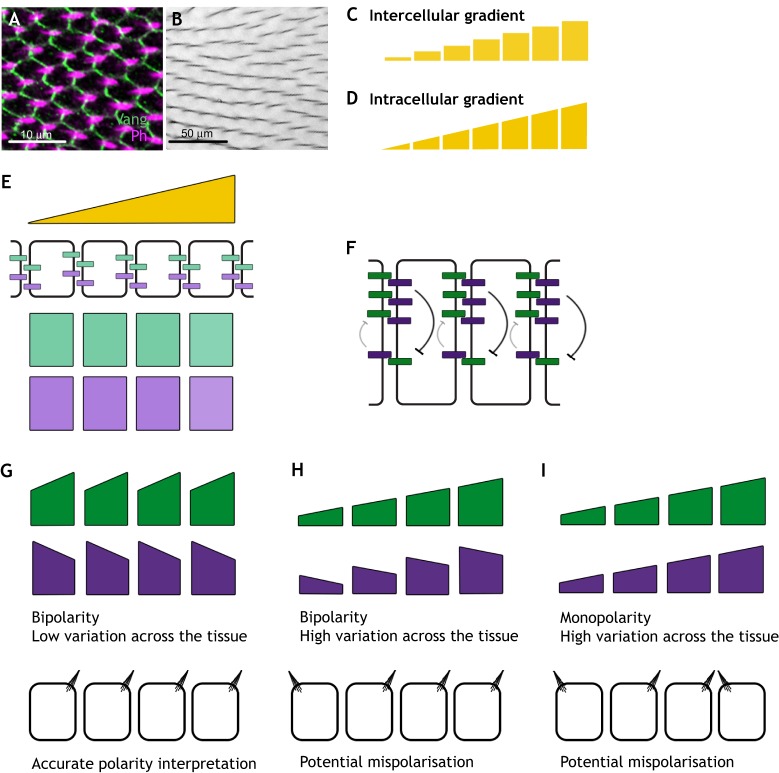
**Gradients in planar polarity specification.** (A) Asymmetric localisation of Vang (green) in the *Drosophila* pupal wing (*w^1118^* pupa at 32 h after prepupa formation) underlies planar polarisation of actin-rich hair placement and orientation, marked by phalloidin (magenta). (B) Adult wing hairs are similarly oriented along the tissue axis. Images are aligned proximal (left) to distal (right) with anterior upwards. Approximate scale bars are shown. (C) Intercellular gradients have varying levels between neighbouring cells. (D) Intracellular gradients vary within individual cells – as well as across the tissue as a whole. (E) Our modelling framework considers a single row of cells (see supplementary information and Box 1 for modelling details). Initial conditions are applied such that unbound molecular bridge components (pale green and pale purple) are uniform across cells, and a gradient is applied across the tissue. Components can move within cells or bind between cells. (F) An imbalance in the concentrations of oppositely oriented complexes at a particular cell junction could be amplified by feedback interactions. In this example, destabilising feedbacks are shown such that the purple component destabilises binding of the green one, leading to the amplification of the localization of the former on that cell edge. (G-I) Three possible steady-state outcomes are shown where bound molecular bridge components (dark green and dark purple) become asymmetrically localised within cells. (G) In the first case, bound components localise to opposite cell ends resulting in bipolarity and each cell shows similar binding levels of components at each end: such low variation in binding levels across the tissue improves the ability of cells to generate a uniform response. (H) In the second case, bipolarity is achieved, but with varying levels of bound protein across the tissue. At the low end of the gradient, noise may lead to errors in the cellular output. (I) In the third case, monopolarity is produced, such that both bound components A and B are enriched on the same side of the cell, with varying binding levels across the tissue. As components A and B are at equal levels at juxtaposed cell edges, this polarity could not be amplified by feedback interactions such as those outlined in F.

Many animal tissues show coordinated polarisation of cells along a planar axis. Such planar polarisation results in the coordinated placement and function of external structures, such as hairs or cilia (Fig. 1A,B), or in coordination of morphogenetic movements (reviewed by Butler and Wallingford, 2017; Davey and Moens, 2017). Underlying such polarised cell behaviours is the subcellular asymmetric localisation of specific polarity proteins, which in turn regulate downstream effectors. Although mechanisms of planar polarity are best understood for *Drosophila* epithelial tissues, numerous lines of evidence support the same principles applying in more complex systems (Goodrich and Strutt, 2011).

In recent years, a general framework for planar polarity specification has emerged (Goodrich and Strutt, 2011; Aw and Devenport, 2017; Lawrence and Casal, 2018). At the top level, global cues exist within tissues, which determine the axis of polarity by biasing protein localisations to one or other side of a cell. Such small biases are then amplified through positive feedback to generate strong polarity (Tree et al., 2002; Amonlirdviman et al., 2005; Le Garrec et al., 2006; Warrington et al., 2017). In parallel, protein complexes between cells, known as molecular bridges, couple cell polarities. This results in smoothing between cells, allowing interpretation of weak or noisy global cues (Ma et al., 2003; Burak and Shraiman, 2009).

A key unresolved issue is the nature of the global cues and how they act to coordinate polarity over extended tissue domains. In some systems, evidence suggests that mechanical forces or cellular rearrangements drive global coordination with the tissue axis (Aigouy et al., 2010; Aw et al., 2016). Furthermore, in many contexts, gradients are known to play a key role in providing polarity cues (reviewed by Lawrence et al., 2007; Strutt, 2009; Aw and Devenport, 2017).

Theories on the role of gradients in developmental biology have moved in and out of fashion for over a century (Wolpert, 1996). Secreted morphogens are now known to be important signals. Generally produced from localised sources, they form concentration gradients as they spread throughout surrounding tissue. Such graded signals can specify cell fate, regulate tissue size and provide directional cues to specify planar polarity (reviewed by Strutt, 2009; Rogers and Schier, 2011; Inomata, 2017). A particular challenge for large tissues is that the steepness of a gradient may be small (e.g. differing between cells by only a few percent). At the top of the gradient, this difference needs to be read against the background of a high overall expression level, while at the low end of the gradient, noise may lead to mispolarisation of individual cells.

In this Hypothesis, we present a theoretical framework to explore the theory that molecular bridges between cells provide a general mechanism by which gradients are interpreted to achieve planar polarisation in animal tissues (see also Struhl et al., 2012; Lawrence and Casal, 2018). A number of previous computational models of planar polarity have incorporated graded polarity cues as providing axis information (e.g. Amonlirdviman et al., 2005; Le Garrec et al., 2006; Abley et al., 2013; Mani et al., 2013; Jolly et al., 2014; Hale et al., 2015); however, there has been no systematic analysis of how gradients can be interpreted by cells during planar polarisation and the role of molecular bridges in this process.

We provide a conceptual basis for understanding how gradients can function in different ways to generate polarised systems. Each gradient mechanism is tested using a generalised and consistent modelling framework, asking how the gradient affects molecular bridge distribution to specify planar polarity across a simple tissue. We do not consider secondary processes, such as feedback amplification of planar polarised protein distributions, or morphogenetic outputs, such as emergence of polarised hairs.

## Gradients in planar polarity – examples and mechanisms

Recent studies, particularly in *Drosophila*, have suggested that concentration gradients can act via a number of different mechanisms to establish directionality (i.e. asymmetric cellular protein distributions) in planar polarity systems. We introduce some examples here and below discuss how they may fit into our proposed theoretical framework.

Directionality in the Fat-Dachsous-Four-jointed (Ft-Ds-Fj) system in the *Drosophila* eye and wing emerges, at least in part, from expression gradients of the Golgi-localised kinase Fj (Zeidler et al., 1999, 2000; Yang et al., 2002; Ishikawa et al., 2008). Fj-dependent phosphorylation of the atypical cadherins Ft and Ds can determine their planar polarised localisation to opposite cell ends, providing a polarity cue (Brittle et al., 2010, 2012; Simon et al., 2010; Ambegaonkar et al., 2012; Bosveld et al., 2012). In the eye, Ds itself is also expressed as a gradient, which may influence directionality in this system (Clark et al., 1995; Yang et al., 2002).

Another well-characterised *Drosophila* planar polarity pathway is known as the ‘core’ pathway and includes the transmembrane proteins Flamingo, Frizzled and Van Gogh (Fmi, Fz and Vang) (reviewed by Goodrich and Strutt, 2011). In the fly wing, the core pathway may be polarised by an extracellular Wnt gradient (Wu et al., 2013). In this system, it has been suggested that gradients of Wnt molecules bind to the Fz receptor generating a gradient of Fz activity. In the developing mouse limb bud, a Wnt gradient has been proposed to establish the planar polarity direction of the homologous pathway, by inducing Vang-like 2 (Vangl2) phosphorylation (Gao et al., 2011; Yang et al., 2017). A Wnt gradient has also been proposed to direct planar polarity in the developing *Xenopus* ectoderm (Chu and Sokol, 2016) and in the mouse inner ear (Dabdoub and Kelley, 2005), although the mechanisms are not yet clear.

### Mechanisms of gradient action and interpretation

In these examples, the mechanisms of gradient action fall into two categories, differing in how extracellular graded signals are interpreted by cells. In the first category, the signal leads to a response at the cellular level. For example, a transcriptional response may specify production of a cellular factor (e.g. Fj or Ds) in proportion to the extracellular signal. Alternatively, the graded signal may lead to activation of a diffusible intracellular factor such as a kinase. In either case, each cell has a different amount of cellular activity from its neighbours, and we refer to it as an ‘intercellular gradient’ (Fig. 1C). In the second category, each cell can directly read and locally interpret an extracellular signal (e.g. a Wnt) that varies across the planar surface of a cell in a localised manner, such that the two sides of the cell read a different level of signal. Even though the graded signal exists extracellularly, at the cellular level we regard this as an ‘intracellular gradient’, because different levels of signal are perceived across the axis of an individual cell (Fig. 1D).

Furthermore, there are two views of how coordinated polarisation across a tissue arises during gradient interpretation (Lawrence et al., 2007; Meinhardt, 2007; Abley et al., 2013). The first mechanism assumes that each individual cell can independently polarise (‘intracellular partitioning’) via intracellular feedback interactions that promote protein sorting within cells. These individual polarities may then be coupled to one another through cell-cell interactions, or coordinated through global signals. It has been proposed that this might represent a conserved element of polarity systems from single cells to plants and animals (Abley et al., 2013). The second mechanism is dependent on cells being intrinsically coupled to polarisable neighbours through molecular bridges. Notably, a common feature of planar polarity systems in animal tissues is the presence of molecular bridges that form between cells; moreover, existing evidence suggests that without the capacity to form such bridges, individual cells cannot themselves polarise (Ma et al., 2003; Lawrence et al., 2004; Matakatsu and Blair, 2004; Chen et al., 2008; Strutt and Strutt, 2008; Devenport and Fuchs, 2008; Struhl et al., 2012).

## A theoretical framework for gradient interpretation by molecular bridges

To illustrate how molecular bridges between cells may interpret a directional cue from a gradient, we first define a computational modelling framework consisting of a single row of cells. Each cell has a pool of molecular components, where each component may represent multiple molecular species in a particular planar polarity system. Components considered are either A only or A and B, which can form molecular bridges between cells. We consider mechanisms based on either homodimer formation (A to A) or heterodimer formation (A to B). Components can localise to the left or right side of each cell, move (i.e. through trafficking or diffusion) between these cell edges or bind reversibly to one another between cells (Fig. 1E). We do not consider the role of unbound components, as their free movement within the cell renders them unpolarised. Ordinary differential equations reflecting the dynamics of these interactions are constructed based on the law of mass action (see Box 1). We apply different gradients to the system, which can drive expression of, or modify, the binding components. We further explore the qualitative effects of varying gradient steepness (see supplementary information).

Box 1. Model formulation **Model equations**

We present a generalised framework based on the model previously described by us in Hale et al. (2015). To convert biochemical reactions into ordinary differential equations, we implement the law of mass action, which is the proposition that the rate of a reaction is proportional to the product of the concentration of reactants. Thus, if we have a chemical reaction
(1)
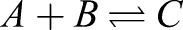

the rate of change of C will be composed of two parts – the formation of C from A and B and destruction of C into its component parts. Thus, the rate of change in the concentration of C is given by the differential equation
(2)
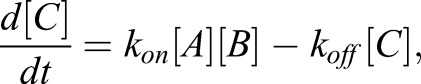

where square brackets indicate concentration of a species and *d*/*dt* indicates a rate of change of some factor over time. The equation is parameterised by the rate constants *k_on_* and *k_off_*.

In our model, complex C represents a molecular bridge formed by binding of components A and B between two cells; therefore, C cannot diffuse or relocalise to another part of the cell without first undergoing a dissociation of binding. However, the individual components A and B are not restricted in this way and can move around the cell. Our modelled tissue is a single row of cells, where each cell has only two compartments, representing a left and a right edge. Thus, in our equations for A and B, we include terms representing this movement. These terms are derived from the finite differences solution to the diffusion equation. Thus, the rate of change in the concentration of A in the left edge of a cell is given by
(3)
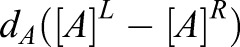

where ^L^ and ^R^ denote the left and right sides of the cell, respectively, and the parameter *d_A_* is the diffusion rate parameter.

For a scheme where A and B bind to one another between two cells to form complex C, which can take either orientation, we can use these methods to convert the set of biochemical reactions of all species in cell *i*:
(4)
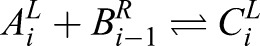

(5)
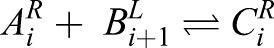

(6)
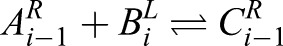

(7)
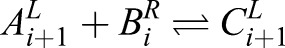

and generate a set of equations to explain the rate of change of all species in cell *i* over time:
(8)
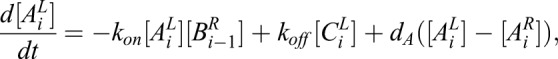

(9)
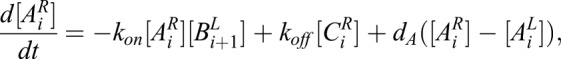

(10)
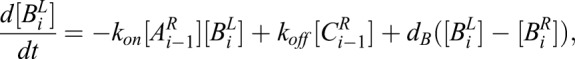

(11)
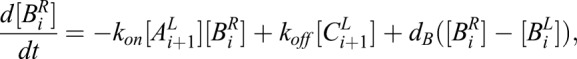

(12)
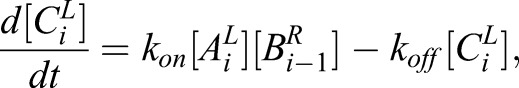

(13)
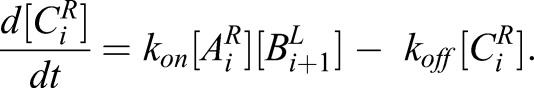

Additional equivalent equations are derived for modified components, such as A*, B* or additional complexes, where appropriate.

**Parameters**

The simple scheme presented here shows four parameters, which will determine the rate of change of the molecular species. These are *k_on_*, *k_off_*, *d_A_* and *d_B_*. The rate constants, *k_on_* and *k_off_*, with units M^−1^ s^−1^ and s^−1^ respectively, determine the rate of complex formation. As we are not using our models to represent specific molecular species, these values are arbitrary, although in schemes with multiple complexes, the relative values become important. This is explained in more detail within the main text.

The unitless diffusion parameters, *d_A_* and *d_B_*, are assumed to be equal for components A and B, and are taken as *d_x_=µ/L^2^*, where *µ*=0.03 µm^2^ is a reasonable estimate for the diffusion coefficient of membrane-associated proteins and *L*=5 µm is the width of each cell (Fischer et al., 2013; Klünder et al., 2013).

A brief comparison of our model with previously published models of planar polarity is provided in Box 2; we also refer the reader to other recent reviews on different modelling efforts (e.g. Axelrod and Tomlin, 2011; Fisher et al., 2017). Most published work has included feedback interactions to amplify initial polarity of complexes. In both the core and Ft-Ds pathways, there is evidence that complexes interact in cis (i.e. in the same membrane compartment in the same cell), either to stabilise similarly oriented complexes or to destabilise oppositely oriented complexes (Fig. 1F, Tree et al., 2002; Cho et al., 2015; Loza et al., 2017; Warrington et al., 2017). Here we consider only the initial interpretation of graded cues in establishing asymmetry of polarity proteins and do not include feedback interactions within our models.

Box 2. Comparison with other models of planar polarity Several groups have modelled planar polarity to aid understanding of the underlying molecular wiring that generates a polarised system. Many of these models built upon experiments on the *Drosophila* core pathway (e.g. Tree et al., 2002), which suggested that an initial directional cue is amplified by feedback interactions to generate a stably polarised system. In the models, these feedback interactions generated what is mathematically known as a bistable system, similar to a Turing pattern formation mechanism.

In such a bistable system, two components act to outcompete one another in a particular location. In the case of planar polarity, these two components are molecular bridges of opposing orientations. For example, in the core pathway model by Amonlirdviman et al. (2005), Pk acted to destabilise Dsh and Fz in the same membrane compartment, leading to the sorting of complexes and an ultimately polarised system. This and similar models were able to simulate outcomes such as hair emergence downstream of the core pathway (Amonlirdviman et al., 2005; Le Garrec et al., 2006; Burak and Shraiman, 2009) or cell division and tissue growth downstream of the Ft-Ds pathway (Mani et al., 2013), as well as reproducing non-autonomous clone phenotypes. However, these models did not focus on the nature of the initial cue and how that cue might ultimately be interpreted by cells to lead to an initial polarity direction.

Our model, which is based on the model of the Ft-Ds pathway presented in Hale et al. (2015) and in some ways similar to the model by Jolly et al. (2014), does not include such feedback interactions. We are only concerned with the initial interpretation of the graded cue to guide asymmetry in complex formation. Although the the published work of Hale et al. and Jolly et al. focuses on specific molecules and gradients, our generalised approach allows us to take a simple system, apply different types of gradients and compare how they are interpreted at the molecular level to drive polarity direction.

We consider a system to be polarised when a higher accumulation of bound component occurs at one side of each cell. However, it is important to note that while a weak initial polarity can subsequently be amplified by feedback or responded to directly by the cell, such amplification or direct response may be confounded by noise. Thus, we consider a fold-difference of bound component between left and right cell edges of at least 2% to be sufficiently polarised above any noise or stochastic variation (e.g. as long known to be sufficient to permit polarised cell migration; Devreotes and Zigmond, 1988).

Here, we describe three possible outcomes, which may lead to different accuracies in response. In the first case, complexes show bipolarity such that two components are enriched on opposite sides of each cell with similar binding levels in each cell (Fig. 1G). We consider this a favourable outcome, as each cell receives a similar strength polarity cue with low variation across the tissue. Our second case (Fig. 1H), while also showing bipolarity, has variation in binding levels across the tissue. These variations may lead to mispolarisation at the low end of the gradient where noise may confound interpretation of the polarity cue. Furthermore, at the high end of the gradient, the difference in levels of bound component across each cell is relatively small compared with the total amount of complexes, again making interpretation difficult. Our final case produces monopolarity (Fig. 1I), such that two bound components accumulate at the same side of the cell and binding varies across the tissue. As for the second case, the effects of noise may confound interpretation of monopolarity at the low end of the gradient and at the high end the amount of polarised material is only a small proportion of the total amount of bound component.

Furthermore, we note that, unlike bipolarity, monopolarity cannot be amplified by feedback interactions between bound complexes. Feedback interactions rely on an initial imbalance between the amounts of oppositely oriented complexes at a particular cell-cell junction (e.g. Fig. 1F). If such an imbalance does not exist, as is the case for monopolarity, neither orientation of complex has an advantage over the other and amplification does not occur. Based on the above considerations, in our assessment of the effects of different gradients on cell polarisation, we seek to identify cases that maximise cell bipolarity, while minimising variations in binding levels across the entire tissue.

## Intercellular gradients

We will first consider intercellular gradients in which the cellular response to an extracellular gradient is a different level of a cellular-wide activity between neighbouring cells (Fig. 1C). In the simplest case, this could reflect the expression level of a component of the molecular bridge, but also encompasses situations where a uniformly expressed bridge component is modified to alter its activity – and the levels of this modification are regulated at a cellular level by the extracellular cue.

### Graded expression of molecular components

Here, we examine cases in which the expression level of the molecular bridge components in each cell is controlled by a graded extracellular ligand. This results in intercellular gradients of the components, with each cell expressing an amount proportional to the level of the ligand. An example is seen in the *Drosophila* eye, where Ds is expressed as a gradient (Clark et al., 1995) and can act as a molecular bridge by binding to Ft in neighbouring cells (Yang et al., 2002; Matakatsu and Blair, 2004). We will consider three cases: (1) homophilic binding of a single component; (2) heterophilic binding, where only one component is graded; and (3) heterophilic binding, where both components are graded.

#### (1) Homophilic binding with graded expression

**Fig. 2.**
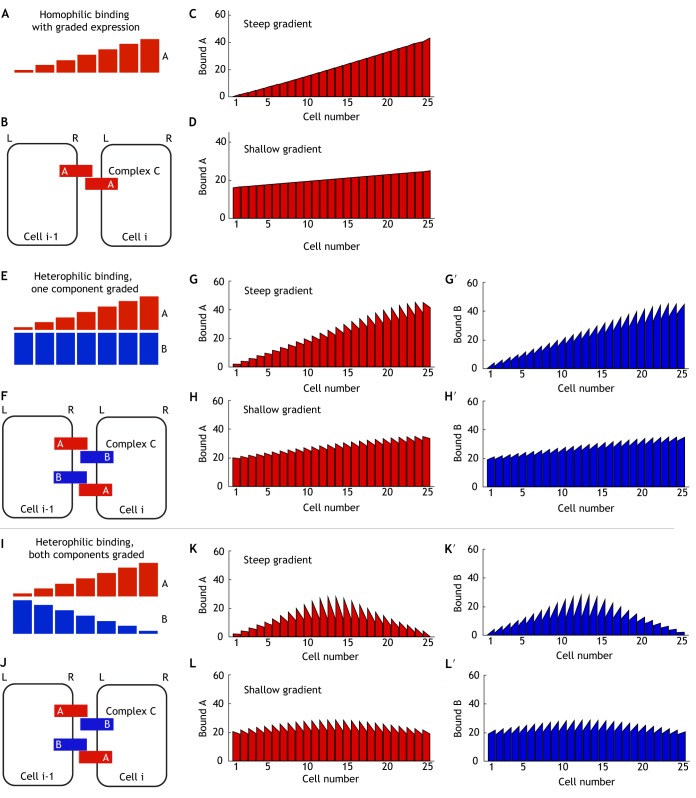
**Polarity establishment by graded expression of molecular bridge components.** (A-D) Model of homophilic binding. (A) Initial conditions – component A is expressed as an intercellular gradient such that each cell expresses a different amount with high levels to the right of the tissue. (B) Complex formation – component A can bind homophilically to itself in neighbouring cells to form complex C. (C) Steady state of simulation showing bound component A levels (i.e. total complex C) at left and right edges of each cell; each cell shows weak asymmetry with component A localising towards cells expressing more binding partner, and total levels of complex vary across the tissue. (D) Steady state of simulation showing bound component A levels – as in panel C but with a shallow gradient as the initial condition. This reduces the variation in levels across the tissue and also reduces cell polarity. As in Fig. 1I, positive feedback cannot amplify the cellular polarity of A. (E-H′) Model of heterophilic binding with one component graded. (E) Initial conditions – component A has graded expression forming an intercellular gradient, whereas component B is uniform across the tissue. (F) Complex formation – components A and B can bind to form complex C in either orientation. (G,G′) Simulation at steady state shows cell bipolarity with bound component A accumulating to the left of each cell (G) and bound component B accumulating to the right (G′); total levels of the complex vary across the tissue. (H,H′) Steady-state simulation with a shallower initial gradient; cell polarity is reduced, while total levels of complex are more uniform across the tissue. (I-L′) Model of heterophilic binding with oppositely graded components. (I) Initial conditions – components A and B are expressed as opposing intercellular gradients, such that component A is highest to the right of the tissue and component B is highest to the left. (J) Complex formation – components A and B bind to form complex C in either orientation. (K,K′) Simulation at steady state shows cell bipolarity with bound component A accumulating to the left of each cell (K) and bound component B accumulating to the right (K′); total levels of complex vary across the tissue. (L,L′) Steady state of simulation with shallower gradient; cell polarity and total levels of complex are more uniform across the tissue.

Each cell expresses a single molecular component, A, as an intercellular gradient (Fig. 2A). Unbound A can redistribute within a cell or bind to A in neighbouring cells to form complex C (Fig. 2B). Allowing simulations to evolve to steady state, we observe a bias in bound A within each cell, such that it accumulates towards the higher end of the gradient where there is more available binding partner (Fig. 2C). The fold-difference across each cell is above our imposed 2% cut-off (Table S1); thus, the system is considered polarised, although binding levels vary across the tissue. A shallower expression gradient of component A reduces the overall tissue asymmetry in levels of binding, but also results in lower cell polarity, with most cells now falling below the 2% fold-difference threshold (Fig. 2D; Table S1). As molecular bridges are homophilic, levels of bound A are intrinsically coupled between cells and only monopolarity results, which cannot be amplified by positive feedback. Thus, we conclude that homophilic binding with graded expression can return only the initial graded information and not actually extract or amplify it further, and is therefore not an optimal mechanism for interpreting molecular gradients.

#### (2) Heterophilic binding – one component is graded

We next examine whether heterophilic binding between components A and B improves polarisation of cells for a given steepness of gradient. In this case, only component A is graded, while B is expressed uniformly across the tissue (Fig. 2E). Components can bind between cells to generate complex C (Fig. 2F), which can form in either orientation (i.e. A-B or B-A). In response to graded expression of A, bound B accumulates to the right side of cells (Fig. 2G′), towards the neighbouring cell with the most available binding partner, with more than a 2% fold-difference across each cell (Table S1). Correspondingly, bound A accumulates to the left of cells (Fig. 2G). When the initial gradient of A is steep (Fig. 2G,G′), there is a large variation in both cell polarity and the overall binding levels across the tissue. However, this variation is reduced by a shallower gradient of A (Fig. 2H,H′) that generates more uniform polarity across the tissue, while still maintaining a fold-difference of over 2% and a much greater difference than the homophilic binding model (Table S1). Furthermore, as cellular asymmetry is bipolar, it may be further amplified by positive feedback. These simulations reveal that heterophilic molecular bridges are better able to maximise cell polarity relative to the steepness of the input gradient (compared with homophilic binding) and represent efficient mechanisms of interpreting molecular gradients to establish polarity.

#### (3) Heterophilic binding – both components are graded

In a case with heterophilic binding between components A and B, if expression of both components were activated by an extracellular ligand, they would have similarly graded profiles, e.g. increasing monotonically from left to right. Heterophilic binding such that A binds B to form complex C would give similar results to the case of homophilic binding, with both bound A and bound B localising towards the high end of the gradients (as in Fig. 2C). This gives weak monopolar asymmetry and varying binding levels across the tissue.

Instead, we consider a case where expression of A is promoted by the gradient, but expression of B is inhibited, to generate opposing gradients across the tissue (Fig. 2I). For simplicity, we assume that the gradient acts with equal strength on both A and B, so the resultant opposing gradients have the same steepness. As before, A can bind B to form complex C in either orientation (Fig. 2J). Bound A and B now accumulate at opposite cell edges, generating strong polarity (Table S1) with respect to overall protein levels (Fig. 2K,K′). Steep gradients of A and B lead to large differences in overall protein levels and cell bipolarity across the row of cells (Fig. 2K,K′). However, shallower gradients of A and B moderate these variations (Fig. 2L,L′), producing relatively even bipolarity across the tissue.

These findings lead us to conclude that: (1) homophilic bridges result in only weak monopolarity for a given gradient input, which furthermore cannot be amplified via positive feedback; (2) heterophilic bridges provide a stronger response to gradient inputs, and can also give rise to bipolarity (that can be further amplified); and (3) shallow molecular gradients are consistent with the formation of more uniform tissue-wide polarity.

### Gradients of protein modification

Some biological examples of gradients that establish polarity appear to act through modification (e.g. phosphorylation) of molecular bridge components, thereby altering their activity as opposed to their expression. An example is the Fj gradient in the *Drosophila* wing (Hale et al., 2015), which acts to phosphorylate its targets within each cell. As Fj has been shown to be functional when tethered in the Golgi (Strutt et al., 2004), this suggests that Ft and Ds molecules are phosphorylated as they pass through the secretory machinery and thus this will occur only once as they are trafficked. Additionally, it is assumed that this modification does not alter their trafficking speed or direction, and thus modification does not inherently generate a cellular asymmetry. We replicate these behaviours in the following simulations. Within this scheme, we will consider three cases: (1) homophilic binding; (2) heterophilic binding, where only one component is modified; and (3) heterophilic binding, where both components are modified.

#### (1) Homophilic binding

**Fig. 3.**
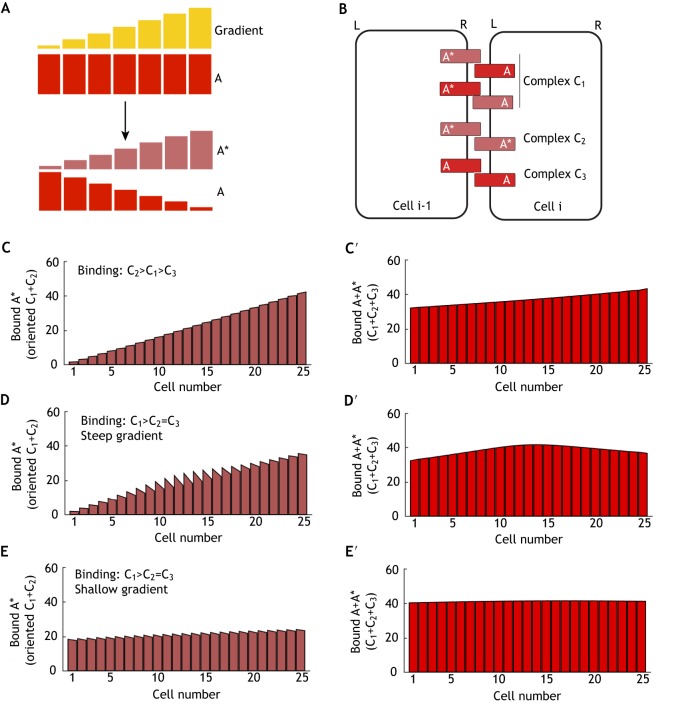
**Polarity establishment of homodimers by an intercellular gradient.** (A) Initial conditions – component A is expressed uniformly, but is modified to form A* by an intercellular gradient. (B) Complex formation – component A, or its modified form A*, can bind between neighbouring cells to form complexes C_1_ to C_3_. Complex C_1_ can form with either orientation. (C,C′) Steady-state simulation result with A* binding to A* (complex C_2_) as the most stable complex. Negligible cell polarity was observed of either bound A* alone (C) or total bound A+A* (C′). (D,D′) In a simulation with A* binding to A (complex C_1_) as the most stable complex, polarisation of bound A* (D), but not total bound A+A* (D′), occurs at steady state; bound protein levels vary strongly across the tissue. (E,E′) In a simulation as in D, but with a shallower gradient, both the cell polarity of bound A* and tissue variation are reduced at steady state (E), although total bound A+A* levels are uniform (E′).

Here, we consider a case in which each cell expresses a single transmembrane protein A, which can be modified to A* in proportion to the gradient (Fig. 3A). Molecular bridges can form between cells through binding of A and A* to form complexes C_1_ to C_3_ (Fig. 3B), where C_1_ (A* binds A) can take either orientation. We assume that the modification occurs on extracellular domains, such that complexes form with different affinities. For example, if we assume that the modification improves binding, then C_2_ (A* binds A*) would be considered the most favoured complex and C_3_ (A binds A) the least favoured. Under these conditions, the steady-state amount of bound A* does not meet the 2% threshold for fold-difference across each cell (Fig. 3C; Table S1). Nor is the 2% threshold for fold-difference met for the total bound A+A* across each cell (Fig. 3C′; Table S1). This is similar to the case outlined above where expression of both A and B is similarly modulated by the gradient.

However, if we consider a case where the modification improves binding between A and A* (e.g. if A is negatively charged, but A* is positively charged), then C_1_ would be the favoured complex. Our simulations reveal that bound A* becomes polarised, with higher levels on the left edge of each cell (Fig. 3D; Table S1). As with heterophilic binding, introducing a shallower gradient reduces the overall tissue asymmetry, but also reduces the strength of cell polarity (Fig. 3E; Table S1). Note, however, that for this polarity to be interpreted, the modification would have to not only alter binding through the extracellular domain, but also induce a conformational change in the intracellular domain such that the cell can sense the difference in A and A*. Conversely, if the cell could not distinguish between A and A*, total bound A+A* results in weak monopolarity (Fig. 3D′,E′).

#### (2) Heterophilic binding – one protein is modified

**Fig. 4.**
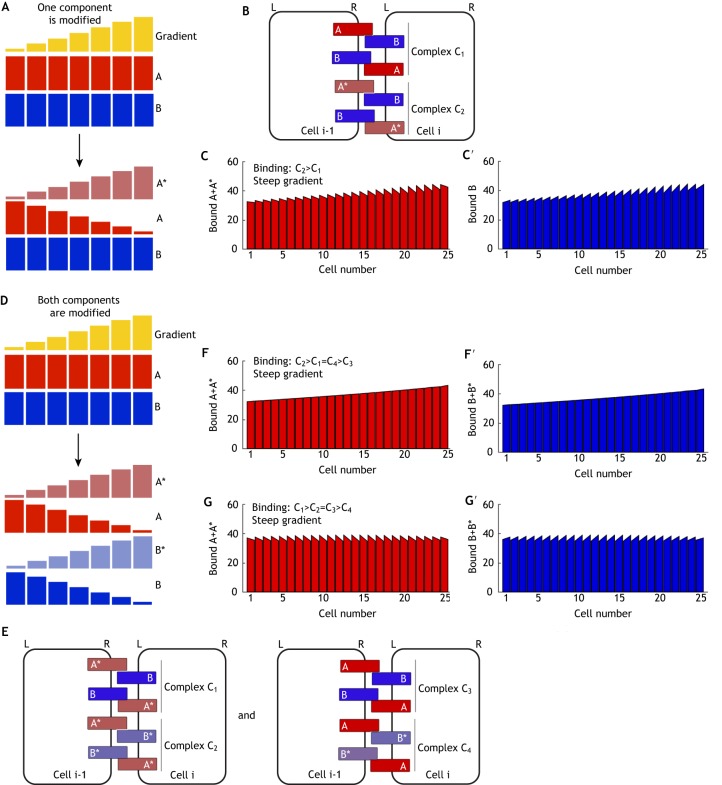
**Polarity establishment of heterodimers by intercellular gradients.** (A-C) Model with one component modified by the gradient. (A) Initial conditions – components A and B are expressed uniformly (top), but component A is modified to form A* by an intercellular gradient, while component B remains uniform (bottom). (B) Complex formation – component A, or its modified form A*, can bind to component B between neighbouring cells to form complexes C_1_ and C_2_, which can form with either orientation. (C,C′) In a simulation with A* binding to component B (complex C_2_) as the most stable complex, the sums of all complexes are plotted at steady state; bound component A accumulates to the left of cells (C) and bound component B accumulates to the right (C′). (D-G′) Model with both components oppositely modified by the gradient. (D) Initial conditions – components A and B are expressed uniformly (top), but are modified to form A* and B* by an intercellular gradient (bottom). (E) Complex formation – component A or A* can bind to component B or B* to form complexes C_1_ to C_4_ (left and right panels), which can form with either orientation. (F,F′) In a simulation with A* binding to B* (complex C_2_) as the most stable complex, negligible cell polarity of bound components A (F) or B (F′) is observed at steady state. (G,G′) In a simulation with A* binding to component B (complex C_1_) as the most stable complex, strong cell polarity of bound components A (G) or B (G′) is observed at steady state with little variation in levels across the tissue.

Here, we assume that each cell expresses uniform levels of A and B, but A can be modified to form A*, according to the gradient (Fig. 4A). Binding can occur to form complexes between A and B (C_1_) or A* and B (C_2_) (Fig. 4B). As with previous examples, we set the modification to be activating such that C_2_ formation is favoured over C_1_. We observe a bipolar distribution such that total bound A+A* accumulates on the left edges (Fig. 4C) and bound B on the right (Fig. 4C′), meeting our 2% threshold for the fold-difference of bound components across each cell (Table S1). Some variation in levels is observed across the tissue. We note that if instead we allow only complex C_2_ to form (or make the strength of binding of C_2_ much greater than that of C1), this variation in levels across the tissue increases substantially, and the result is now as seen in Fig. 2G,G′. Therefore, allowing additional binding to form complex C_1_ increases the amount of complex in cells at the left end of the tissue, which balances the variation across the tissue that would be observed should only C_2_ form (Fig. S1A,B). We note that as the cellular asymmetry is bipolar, it can be further amplified by positive feedback.

#### (3) Heterophilic binding – both proteins are modified

We next consider a case where each cell expresses components A and B, both of which can be modified according to the gradient to form A* and B* (Fig. 4D). Binding leads to the formation of four possible complexes, C_1_ to C_4_ (Fig. 4E), in either orientation. If we assume that the modification enhances binding of both A and B to each other, then C_2_ is the most favourable complex. This results in weak asymmetry of bound A and B, which is also monopolar, even when the gradient is steep (Fig. 4F,F′). This does not meet our 2% threshold for the fold-difference of bound components across each cell (Table S1). However, if we assume that modification by the gradient has opposing effects on A and B, e.g. it enhances binding of A, but inhibits binding of B, then complex C_1_ would be the most favoured. To illustrate this, we ran simulations where C_1_ was the most favoured and found that bound A and B showed bipolarity, meeting the 2% threshold in fold-difference (Table S1), with approximately uniform levels across the row of cells, even with a steep gradient (Fig. 4G,G′).

In this scenario, if only complex C_1_ can form, the outcome resembles that seen in Fig. 2K, in which A and B are expressed in opposing gradients and form a heterodimer (Fig. 2I). In the earlier model, a shallower gradient allows more homogenous polarity across the tissue (Fig. 2L,L′). In the current model, the additional complexes C_2_, C_3_ and C_4_ create more uniform polarity across the tissue, even if the gradient is steep. It becomes clear why this is the case if we consider the steady-state plots of each individual complex (Fig. S1C-F). In particular, complexes C_2_ and C_3_ bind with much higher levels at the edges of the tissue (Fig. S1D,E). When acting in combination with other complexes, overall tissue variation in cell polarity is reduced.

### Summary of analysis of intercellular gradients

In summary, when considering the interpretation of intercellular gradients to generate polarity, heterodimeric bridges between cells are more effective than homodimeric bridges. However, we note that the performance of homodimeric bridges is improved if the gradient modifies component A such that binding is favoured between modified and unmodified components (e.g. A*+A), thereby generating a heterodimer-like system (Fig. 3D,E). Minimally, the system requires that at least one component of the molecular bridge becomes graded or is modified according to a gradient.

Moreover, in heterodimer systems where only one complex forms, shallower gradients reduce the variation in binding levels across the tissue, but this comes at the cost of reduced cell polarity (e.g. Fig. 2G versus Fig. 2H, Fig. 2K versus Fig. 2L). However, relatively homogeneous tissue polarity can be achieved with a steeper gradient if multiple complexes bind (e.g. Fig. 2G versus Fig. 4C, Fig. 2K versus Fig. 4G).

Finally, in heterodimer systems, it is important that the gradient only modifies one component or has opposite action on each component. This ensures that bipolarity (rather than monopolarity) is produced, which can then be amplified via positive feedback.

## Intracellular gradients

We will now consider intracellular gradients, which differ across the axis of each cell (Fig. 1D). We discuss two possible scenarios: the first invoking local modification of one of the molecular bridge components; and the second involving direct modulation of bridge formation by a locally acting cue.

### 

#### (1) Local modification of A to A*

**Fig. 5.**
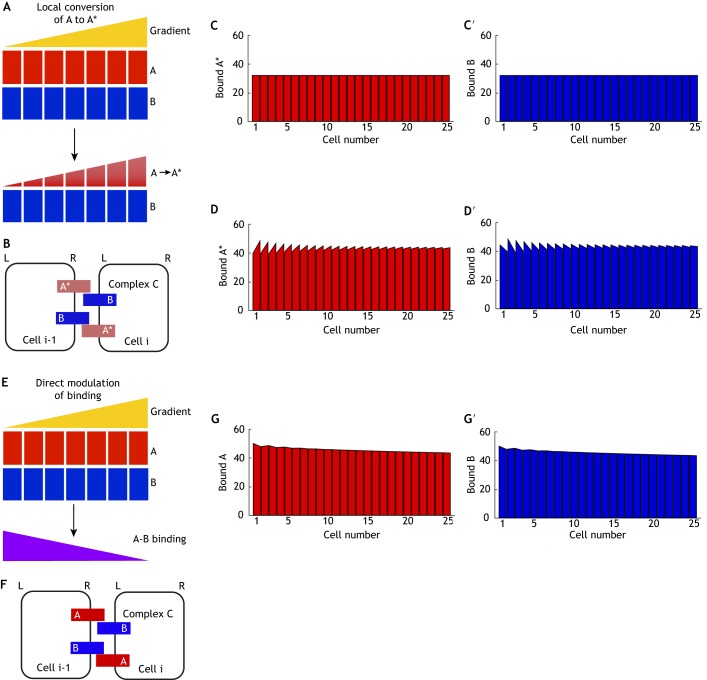
**Polarity establishment by intracellular gradients.** (A-C′) Model of local conversion of component A to A*. (A) Initial conditions – components A and B are expressed uniformly (upper), but A is continually locally converted to A* by an intracellular gradient, while component B remains uniform (lower). (B) Complex formation – A* can bind to B between neighbouring cells to form complex C, which can take either orientation to that shown. (C,C′) In a simulation where diffusion of A* is the same as that of component A, bound A* becomes uniform and no cell polarity is observed at steady state. (D,D′) In a simulation where A* is unable to diffuse and thus accumulates in response to the gradient, complexes form with biased orientation at steady state such that bound A* accumulates to the right of cells (D) and bound B to the left (D′). (E-G′) Model of direct inhibition of binding. (E) Initial conditions – binding between components A and B is inhibited by an intracellular gradient. (F) Complex formation – component A can bind to B between neighbouring cells to form complex C, which can take either orientation. (G,G′) In a simulation where binding is inhibited according to a gradient, negligible cell polarity is generated at steady state, as the gradient does not distinguish between orientations of complex C and thus does not promote accumulation of either orientation.

We first consider a case where the gradient induces local modification of A to form A* (Fig. 5A). This could be through ligand-receptor binding or local post-translational modification. Thus, if the polarising cue is at a higher level at one side of the cell, more A* will be produced at that side. As we assume that this mechanism is acting at the cell surface rather than in a sub-cellular compartment, we simulate this as acting continuously. We allowed A* (but not A) to bind to B (Fig. 5B), and found that complexes do not become polarised (Fig. 5C,C′; Table S1). As A can move within the cell, and is continually converted to A*, at steady state almost all of component A has become A* and thus has similar levels in each cell and is symmetrically distributed.

However, if we consider an alternative case where modification of A to A* stops its ability to redistribute to another part of the cell, bound A* accumulates towards the higher end of the gradient (right cell edge; Fig. 5D) and bound B accumulates towards the lower end of the gradient (left cell edge; Fig. 5D′), resulting in bipolarity that meets our imposed 2% threshold (Table S1). A similar result was observed in a model of intracellular partitioning, which also resulted in A* localising towards the higher end of the gradient (Abley et al., 2013), due to positive feedback interactions which locally reinforce A* localisation.

It is interesting to note that the intracellular gradient shown here produces an opposite polarity to an intercellular gradient (e.g. Fig. 4A-C). The intracellular gradient relies on A* accumulating on the side of the cell exposed to the higher level of the gradient. In this case this drives the localisation of bound A* to the right and bound B to the left. However, as intercellular gradients are driven by differences between cells, such gradients cause B to localise towards neighbouring cells on the right, where more of its most favoured binding partner is found (see Fig. 4). Thus for a given system, it may be possible to predict the mode of action of a graded cue (activating or inhibiting) if the polarity direction and nature of the gradient (intracellular or intercellular) are known.

#### (2) Direct modulation of binding

A further proposed mechanism of intracellular gradient action is generation of polarity via direct inhibition of molecular bridge formation, such as through a gradient of Wnt molecules as proposed in the *Drosophila* wing (Wu et al., 2013). Here, we examine this through simulations where we allow a gradient to act at each junction to modulate binding between components A and B (both of which are uniformly distributed across the tissue) (Fig. 5E,F). However, as complex C is able to form in either orientation at a particular junction, both orientations are altered equally by the gradient and neither is favoured. In our simulations, bound A and B become only weakly polarised in each cell, failing to meet the 2% fold-difference threshold (Fig. 5G,G′; Table S1). Asymmetry is monopolar and thus cannot be amplified by positive feedback. We note that were the graded molecule to promote complex formation, the overall tissue asymmetry would be reversed (i.e. there would be more binding on the right), but bipolarity would still not be generated. Therefore, we suggest that this is not a viable mechanism for cells to interpret molecular gradients (though see below for further discussion of potential modes of Wnt action).

#### Summary of analysis of intracellular gradients

In summary, an intracellular gradient can effectively promote planar polarity through generation of local accumulation of a polarity component. This may be through limiting its movement, as suggested here, or via local stimulation of self-enhancing feedback interactions as previously suggested (Abley et al., 2013). However, mechanisms that directly affect binding between heterodimeric bridge components result in only weak monopolar cellular asymmetries. Moreover, as for intercellular gradients, to establish bipolarity the gradient must act on only one binding component or have opposing effects on each component.

## Biological examples

To further illustrate how molecular bridges can interpret graded cues, we discuss our framework in the context of known examples from animal tissues. In each case we generate diagrams of hypothesised cell polarity based on our previous simulated results.

### Fat-Dachsous polarity in the *Drosophila* wing

One of the best-characterised examples of a gradient-reading polarity system is the Ft-Ds-Fj pathway in *Drosophila*. Ft and Ds are atypical cadherins that bind to one another heterophilically between cells (Ma et al., 2003; Matakatsu and Blair, 2004) and become asymmetrically localised to opposite sides of a cell (Ambegaonkar et al., 2012; Bosveld et al., 2012; Brittle et al., 2012). Fj is a Golgi-localised kinase that phosphorylates the extracellular domains of Ft and Ds (Strutt et al., 2004; Ishikawa et al., 2008). *In vitro* studies suggest that this phosphorylation modulates binding affinities between Ft and Ds, by inhibiting Ds binding to Ft, but enhancing Ft binding to Ds (Brittle et al., 2010; Simon et al., 2010). These findings have been supported *in vivo*, using fluorescence recovery after photobleaching (FRAP) measurements as a proxy for strength of heterodimer binding in the *Drosophila* wing, as well as with computational modelling based on mass-action binding kinetics (Hale et al., 2015).

**Fig. 6.**
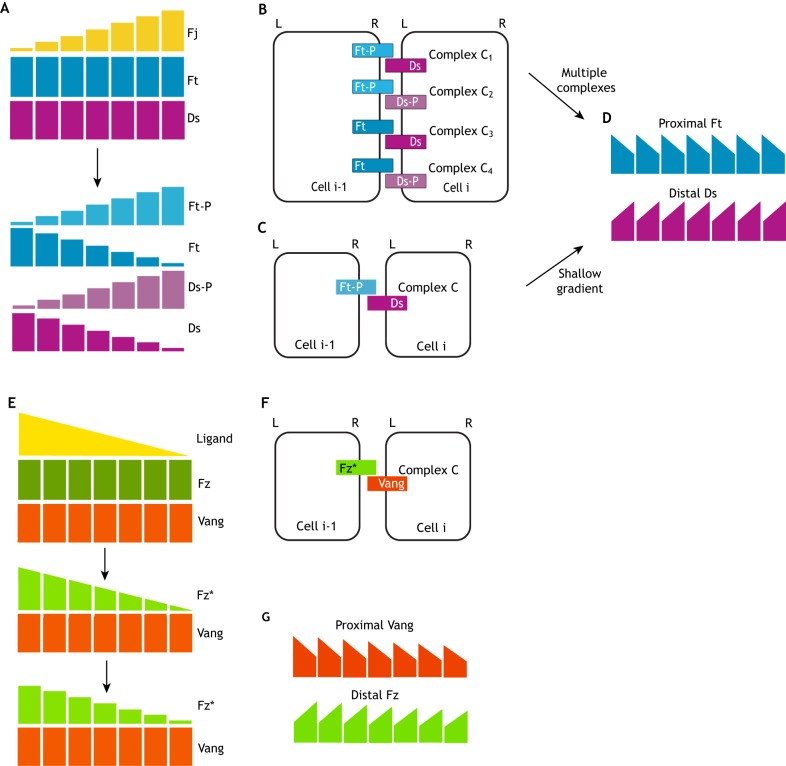
**Biological examples of gradients in polarity establishment.** (A-D) Ft-Ds polarity is established by a Fj gradient in the *Drosophila* wing. (A) Initial conditions – Fj is expressed as an intercellular gradient and can phosphorylate extracellular domains of Ft and Ds to generate gradients of phosphorylated Ft and Ds (Ft-P and Ds-P, respectively). (B,C) Complex formation – we consider that either four complexes can form between Ft and Ds molecules in neighbouring cells: with complex C_1_ having the highest affinity (B) or only complex C_1_ forming at detectable levels (C). All complexes can form in either orientation shown. (D) Our modelling predicts that either case can result in Ft-Ds polarisation, although C requires a shallow gradient to avoid strong variation in binding levels across the tissue. (E-G) The Le Garrec et al. (2006) computational model of Fz-Vang polarity is established by a Fz* activity gradient, generated by ligand binding. (E) Initial conditions – an extracellular ligand binds to Fz to generate an intracellular gradient of Fz*. As Fz* can diffuse, we propose that this would generate an intercellular gradient of Fz* across the tissue (bottom panel). (F) Complex formation – Fz* and Vang can bind to form a complex (in either orientation to that shown), although Fmi could also be involved in forming this molecular bridge. (G) Our modelling predicts that the system generates proximally localised Vang and distally localised Fz.

The model of Hale et al. (2015) is comparable with the model shown here, in which binding is heterophilic and both proteins are modified by the gradient (Figs 4D and 6A). In both models, we suggest that when all four possible complexes form and C_1_ (Ft-P-Ds) is the most favoured (Fig. 6B), homogeneous polarity and protein levels can be generated across the tissue even in the presence of a steep gradient (Fig. 6D). However, should complex C_1_ be the only complex to form (resembling Fig. 2I), as considered in previous computational models (Mani et al., 2013; Jolly et al., 2014), homogenous polarity can also be produced if the gradient is sufficiently shallow (Fig. 6C,D). A less steep gradient reduces cell polarity (Fig. 2L compared with Fig. 2K), but as asymmetry is bipolar, this could be additionally amplified by feedback interactions.

In the fly notum at pupal stages, the Fj gradient runs over fewer cells than in the developing wing, potentially generating a steeper gradient (Bosveld et al., 2012): notably, Ds polarity appears most evident at the mid-point along this Fj gradient. This is consistent with a scenario that combines a steep gradient with a primary complex (Ft-P and Ds) that is much more stable than other combinations of heterodimer, similar to that shown in Fig. 2K,K′. However, this remains to be formally tested *in vivo*. Interestingly, if alternatively Fj acted as a secreted molecule forming an intracellular gradient – as was previously proposed on its initial discovery (Villano and Katz, 1995; Brodsky and Steller, 1996) – our modelling predicts that Ft-Ds polarity would be reversed.

### Core polarity in the *Drosophila* wing

The core planar polarity pathway has been most extensively studied in the *Drosophila* wing. Fmi is a cadherin that can homodimerise between cells (Chae et al., 1999; Usui et al., 1999). Transmembrane proteins Fz and Vang bind to Fmi, but in apposing cell membranes, and stabilise its dimerisation, forming an inherently asymmetric molecular bridge (Usui et al., 1999; Strutt, 2001; Bastock et al., 2003; Strutt and Strutt, 2008). Additionally, there is evidence that Fz and Vang can bind intercellularly (Strutt and Strutt, 2008; Wu and Mlodzik, 2008; Wu et al., 2013), although their junctional localisation is dependent on Fmi (Bastock et al., 2003).

Manipulations of *fz* levels – which cause hair polarity defects in the wing and ommatidial polarity defects in the eye – suggest either that there may be a gradient of Fz activity across the tissue or that Fz may be acting in a cell-cell relay to convey polarity information (Vinson and Adler, 1987; Zheng et al., 1995; Adler et al., 1997). More recently it was suggested that an activity gradient in the wing was caused by a gradient of Wnt molecules, namely Wingless (Wg) and Wnt4 (Wu et al., 2013). This mechanism was proposed to act via Wg/Wnt4 inhibiting binding between Fz and Vang in a dose-dependent manner. However, our models predict that simply inhibiting binding would not generate a bipolar polarity cue, as both orientations of the complex would be affected by the Wnt gradient (Fig. 5E-G).

Nevertheless, previous computational modelling has demonstrated that a Fz activity gradient could be sufficient to direct polarity (Le Garrec et al., 2006). In this model, Fz is converted to Fz* by an activating ligand gradient running from proximal to distal (i.e. opposite to the observed Wg/Wnt4 gradient; Fig. 6E-G). Fz* then competes intracellularly with Vang for Fmi binding (while Fz is unable to bind Fmi). Fz*-Fmi and Vang-Fmi complexes bind across a cell junction to form the asymmetric tetramer complex Fz*-Fmi:Fmi-Vang (intercellular junctions denoted here by ‘:’). As individual molecules and unbound Fz*-Fmi and Vang-Fmi complexes can diffuse within cells and Fz* does not return to a Fz state, we predict that this model generates an intercellular gradient of Fz* (Fig. 6E, bottom). This is comparable to our model shown in Fig. 4A-C, where one molecule is modified (A*/Fz*), except with the gradient running in the opposite direction. This would result in bound Fz* (A*) accumulating on the right and bound Vang (B) accumulating on the left (Fig. 6F,G, reversed polarity when compared with Fig. 4C,C′ due to reversal of the gradient).

It further follows that the observed distal to proximal Wg/Wnt4 gradient in the wing could act as an effective planar polarity cue for the core pathway if two conditions hold: (1) the default state of Fz is ‘active’ (i.e. Fz*, able to bind Fmi) and Wg/Wnt4 binds to Fz* and converts it to Fz (thus inhibiting its ability to bind to Fmi and form a molecular bridge); and (2) this binding/modification is stable and retained as Fz redistributes within cells. This is again equivalent to the model shown in Fig. 6E-G, where an inactivating Wnt gradient from distal to proximal results in distal Fz accumulation and proximal Vang accumulation.

### Core polarity in vertebrate limb bud

The core polarity pathway in the vertebrate limb bud has also been suggested to derive its directional cue from a Wnt gradient. However, in this case it is proposed that Wnt5a ligand binds to Ror2 receptors, which can form a complex with Vangl2 and induce Vangl2 phosphorylation (Gao et al., 2011; Yang et al., 2017). It is assumed that an intercellular gradient of Vangl2 phosphorylation is generated across the tissue with highest levels at the distal tip of the limb bud. This is comparable with our model in Fig. 4A-C where the gradient acts once on one component of the complex, which can then redistribute within each cell. In this case, A would represent Vangl2, A* would represent phosphorylated-Vangl2 and B would represent the other side of the molecular bridge complex, likely containing a Fz molecule. In our model, bound A accumulates on the proximal (left) edges of cells, and so recapitulates Vangl2 polarity. As this model uses an activating gradient, where A*-containing complexes are more stable than A-containing complexes, this suggests that Wnt5a would likely play an activating role and this is consistent with experimental evidence (Gao et al., 2011).

Recent studies have shown that *Drosophila* Vang is phosphorylated in the wing and this phosphorylation is apparently required for asymmetry of complexes (Kelly et al., 2016). It would be interesting to test whether this phosphorylation also occurs in response to a Wnt gradient, as occurs for Vangl2 in the limb, and whether Wnt may thereby use this mechanism to provide directionality to core polarity in the wing.

## Concluding remarks

This Hypothesis explains how systems of molecular bridges make efficient gradient-reading systems to generate planar polarity. We have carried out a systematic analysis, providing a conceptual basis for how different mechanisms of action by gradients could provide directional information. Our simulations lead to testable predictions about how activating or inactivating intracellular or intercellular gradients may influence polarity direction.

We draw three important conclusions. First, systems of heterodimers are more efficient than homodimers at extracting graded information. In particular, in cases where the graded cue affects the activity of only one component of a heterodimer system, or affects the two components oppositely, then this results in bipolarity where the components accumulate at opposite cell edges (e.g. Fig. 2G,K). This is significant, because bipolarity can be further amplified by positive-feedback interactions, whereas monopolarity cannot.

Second, while steeper gradients lead to stronger cell polarity, they can also lead to significant variations in levels of complex binding and cell polarity across the tissue (e.g. Fig. 2G,K). However, more uniform polarity across tissues can be achieved by the addition of complexes forming between components with different tissue profiles (e.g. Fig. 4C,G). Such systems appear better suited to generating homogeneous polarity in the context of varying gradient steepness, as may occur during tissue growth. Third, we note that intracellular and intercellular gradients lead to opposite cell polarity, as has been discussed previously (Aw and Devenport, 2017).

Importantly, our work suggests that measuring the profiles of cell polarisation for different planar polarity systems across different tissues may provide an important tool for predicting both the direction and likely mechanism of action of any gradient in play. In the section on ‘Biological examples’ we summarise the predictions that our work makes with regard to well-studied planar polarity systems. In particular, we also make predictions regarding possible modes of Wnt gradient activity in planar polarity, which has been a subject of much debate. Finally, we note that the flexibility of heterodimer systems in reading gradient information makes it possible that the same system may read different gradients in different contexts.

## Supplementary Material

Supplementary information
