# Supplementary material for: A theoretical framework for planar polarity establishment through interpretation of graded cues by molecular bridges
Source: Development. 2019 Feb 1;146(3):dev168955. doi: 10.1242/dev.168955 (PMC6382004; doi:10.1242/dev.168955)
Supplement: Supplementary information [file develop-146-168955-s1.pdf]

Supplementary Information: Model Formulation

Tissue geometry and boundary conditions

Tissue is represented by a one-dimensional row of 25 equally sized cells, each with two compartments representing left (^{L}) and right (^{R}) cell membranes. We impose zero-flux boundary conditions through the addition of a single membrane compartment at either end of the cell row, allowing binding components to form complexes at the ends of the row, but not diffuse (Fig.1E). These additional half-cell compartments are omitted from the graphs of simulation results. We assume that protein dynamics and complex formation occurs on a timescale faster than changes in cell rearrangements or divisions. Therefore the tissue architecture remains unchanged throughout our simulations.

Protein dynamics

We simulate mass-conserved pools of molecular components, A and B, localising within each cell, with arbitrary units of concentration. As for many previous models of planar polarity, we assume that polarisation occurs on a faster timescale than new protein synthesis rendering our assumption of mass-conversation valid. However, for systems where protein turnover occurs on a faster timescale than complex sorting, we note that our models may no longer give a representative view of *in vivo* reality.

In our simulations each component may represent multiple protein species in a particular planar polarity pathway. For example, 'A' may represent Fat in the Fat-Dachsous pathway, or a combination of Frizzled, Dishevelled and Diego in the core pathway. Within each full cell, molecular components can localise to left and right compartments and move between them through diffusion or trafficking. Molecular components can also bind to form complexes between neighbouring cells. Thus in the case of heterodimer formation, the following reversible binding reaction can occur at each cell-cell interface:

$$A + B^{\#} <-> C, \qquad (1)$$

Development • Supplementary information

where # refers to a species in a neighbouring cell and C represents the complex formed. In the case of homodimeric complex formation, component ‘A’ replaces ‘B’. Complexes of the reverse orientation can also form at the same interfaces, according to the following reaction:

$A^{\#} + B \leftrightarrow C^{\#}$. (2)

We explain the formulation of our ordinary differential equations representing mass-action kinetics and diffusion, along with associated parameters, in Box 1 of the main text.

Initial conditions

Since our models represent generalised systems rather than specific molecular pathways, our initial conditions are somewhat arbitrary. However, we have applied what we think is a reasonable ballpark of *in vivo* gradient steepness. For example, the Fj gradient has been estimated as having a 3% difference between cells in the *Drosophila* wing disc (Hale et al, 2015) which falls at the steeper end of our simulated gradients in Figures 3 and 4.

Models with graded molecular components – Figure 2

Each cell is initialised with an amount of each molecular component that is uniformly distributed within each cell, but differs between neighbouring cells to form an intercellular gradient (Fig.1C). For simplicity, gradients are considered to be linear such that levels in each cell vary by the same absolute amount. For example in Fig.2C, initial levels of component A vary from 0 to 50 units over 27 cells (including the two half-cells at the ends of the row); therefore each cell differs by 1.92 units of A. This is considered to be a ‘steep’ gradient. Where ‘shallow’ gradients are simulated, components vary from 20 to 40 units and thus each cell differs by 0.77 units.

Models with a graded molecule that modifies molecular components – Figures 3 and 4

Each cellular compartment is initialised with 50 arbitrary units of each molecular component. A graded molecule then acts to modify components such that a proportion of the component is converted to a * form. We consider

Development • Supplementary information

such gradients to act with a particular percentage activity. For example, a 'steep' gradient is considered to run from 0% to 100% activity such that 0% or 100% of molecular component is converted to the * form, at either end of the row of cells. This is the case in Fig.3D, where each cell differs by 3.84% activity of the graded molecule. A 'shallow' gradient is considered to run from 40% to 60% activity and thus each cell differs by 0.77% activity of the graded molecule.

Models with cellular gradients – Figure 5

Each cellular compartment is initialised with 50 arbitrary units of each molecular component. A graded molecule varies continuously across the row of cells such that left and right compartments within each cell have differing levels, but abutting junctions of neighbouring cells have the same levels (Fig.1D). The gradient either modifies components to their * forms (Fig.5C,D), as previously described, or directly alters binding of complexes at specific junctions (Fig.5G). In the latter case, the governing equations are altered such that k_{off} parameters are multiplied by the gradient function $g(x)$, which varies linearly with spatial position (x).

Parameters

The parameters in our models are the binding rate constants ($k_{on,j}$) and unbinding rate constants ($k_{off,j}$) and diffusion coefficients (d_n). Here 'j' specifies the complex number, for example in the model shown in Figure 3, j will take values 1-3 since three possible complexes can form. We use 'n' to denote the molecular species, for example in Figure 3, n could refer to A or A*. In all simulations, we set $k_{on,j}$ and d_n values to 1, unless otherwise stated. In general varying d_n over two order of magnitude does not alter the stable steady state, but only the timescale over which it is reached. Subtle differences may be observed if different molecular components have varying diffusion rates, however this has not been explored here.

In simulations with only one possible complex forming (Fig.2; Fig.5), k_{off} is set to 1. In simulations with multiple complexes (Fig.3; Fig.4), k_{off} values are set

Development • Supplementary information

with relative levels in mind, to determine which complex is most favoured.

These are listed here:

Figure	Panel	Complexes	k_{off}
Fig.3	panel C,C'	C_1, C_2, C_3	5, 1, 10
	panel D,D',E,E'	C_1, C_2, C_3	1, 10, 10
Fig.4	panel C,C'	C_1, C_2	5, 1
	panel F,F'	C_1, C_2, C_3, C_4	5, 1, 10, 5
	panel G,G'	C_1, C_2, C_3, C_4	1, 5, 5, 10

Table S1 - Polarity quantitation for simulations

Figure	Original gradient (difference between cells)	Ave absolute difference (highest edge - lowest edge) [a.u.]	Ave fold difference ([highest edge - lowest edge] / lowest edge)*100 [%]	Number of cells with < 2% asymmetry [max 25]
2C	1.92	1.62	17.22	0
2D	0.77	0.36	1.72	21
2G	1.92	3.73	14.97	0
2H	0.77	1.77	6.26	0
2K	1.92	7.09	80	0
2L	0.77	5.42	27	0
3C	3.84%	0.67	4.75	2
3C'	3.84%	0.44	1.2	24
3D	3.84%	3.29	20.6	0
3E	0.77%	1.4	7.06	0
4C	3.84%	1.95	5	0
4F	3.84%	0.45	1.2	25
4G	3.84%	3.35	9.55	0
	**Original gradient (difference across cells)**			
5C	3.08%	0	0	25
5D	3.08%	3.11	7.58	1
5G	4%	0.27	0.58	23

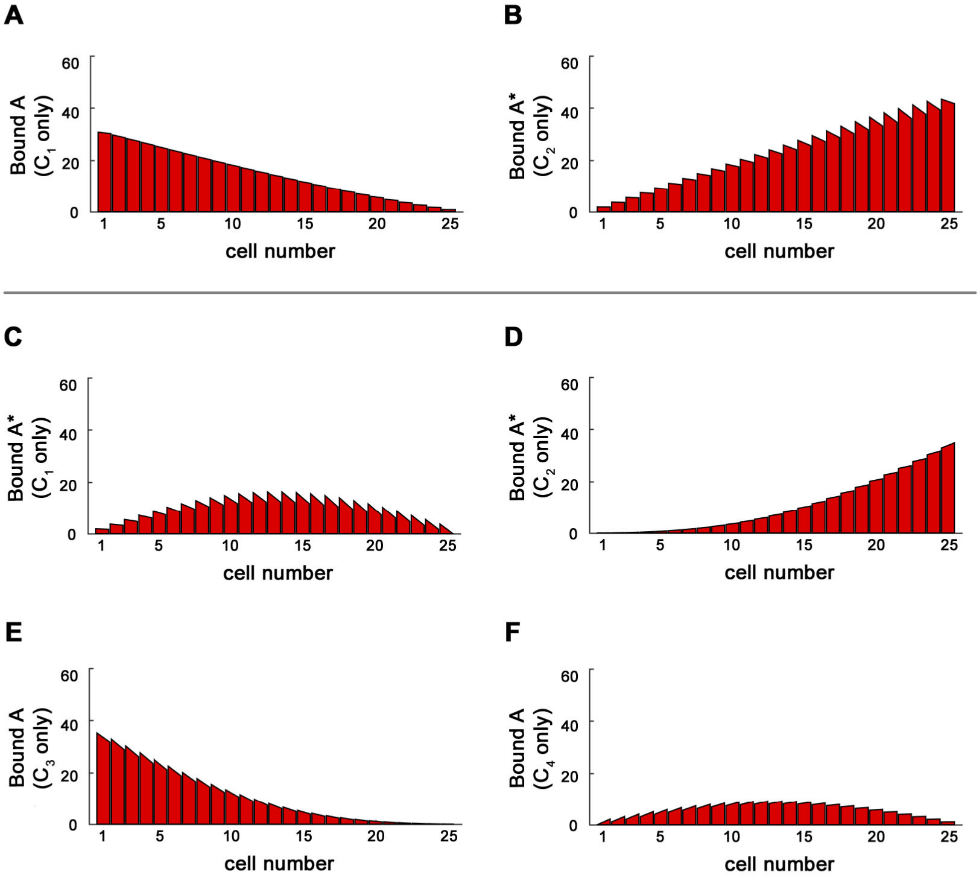

Figure S1. Additional complexes contribute to reduced variation across the tissue

(A,B) As shown in Fig.4C, summing multiple complexes leads to low variation in binding levels across the tissue. Here we plot bound A/A* from individual complexes (C_1 and C_2). Complex C_2 shows higher levels at the left end of this tissue. When summed with complex C_1, variation across the tissue is reduced.

(C-F) As shown in Fig.4G, summing multiple complexes leads to low variation in binding levels across the tissue. Bound A/A* from individual complexes (C_1 to C_4) are plotted. While complexes C_1 and C_4 are limited by the low levels of a particular binding partner at both edges of the tissue, C_2 and C_3 show high levels of binding at either end, resulting in an overall even distribution.

Development • Supplementary information
